# Prevention Is the Best Strategy to Solve Coastal Eutrophication

**DOI:** 10.1021/acs.est.5c02456

**Published:** 2025-05-08

**Authors:** Kentaro Hayashi, Hirotsugu Arai, Yu Umezawa, Takashi Onodera, Kazuyo Matsubae, Keisuke Koba

**Affiliations:** † Program Research Department, Research Institute for Humanity and Nature, National Institutes for the Humanities, Kyoto 603-0847, Japan; ‡ Department of Environmental Science on Biosphere, 13125 Tokyo University of Agriculture and Technology, Fuchu 183-8509, Japan; § Regional Environment Conservation Division, 13585 National Institute for Environmental Studies, Tsukuba 305-8506, Japan; ∥ Graduate School of Environmental Studies, 13101 Tohoku University, Sendai 980-8572, Japan; ⊥ Center for Ecological Research, 83942 Kyoto University, Otsu 520-2113, Japan

**Keywords:** eutrophication, oligotrophication, nitrogen, phosphorus, enclosed sea

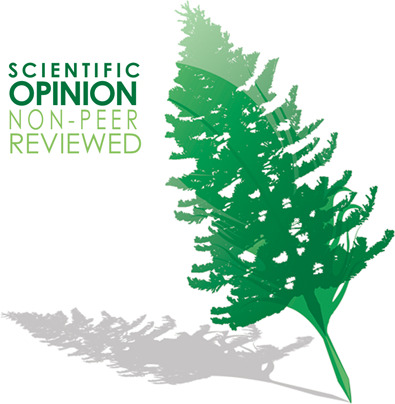

## Graphical abstract

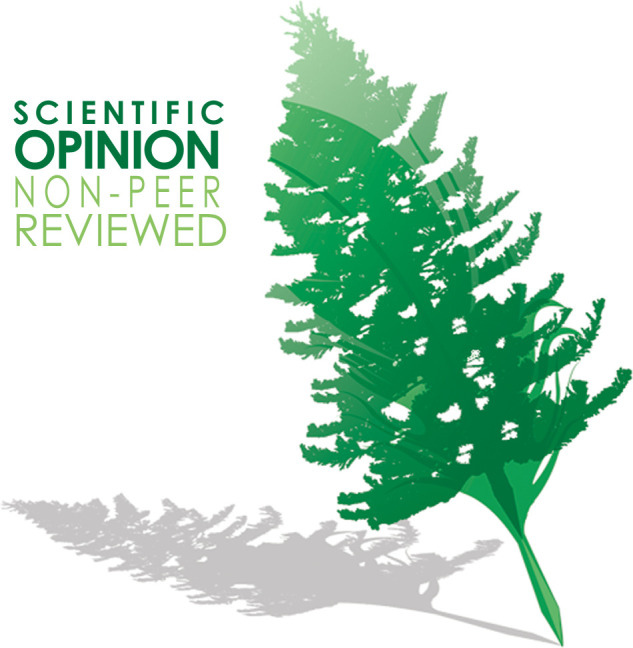

Human activities such as agriculture and urbanization increase nutrient inputs, primarily nitrogen and phosphorus, into catchments, contributing to eutrophication in downstream lakes and enclosed seas. Planetary boundary studies have shown that human-induced disruptions to the nitrogen and phosphorus cycles have resulted in exceedances to Earth system limits with respect to coastal eutrophication.^1^ In 2020, the United Nations Environment Programme (UNEP) established the Working Group on Nitrogen (WGN) based on the first “Resolution on Sustainable Nitrogen Management”, which was adopted at the 4th United Nations Environment Assembly in 2019. The WGN promotes international cooperation on nitrogen management to mitigate the threat of nitrogen pollution while preserving the benefits of nitrogen use. It also encourages member countries to develop national action plans. Notably, Japan published its action plan in September 2024.^2^ The Global Partnership on Nutrient Management, a multistakeholder group for which UNEP serves as the secretariat, also aims to promote activities for sustainable nitrogen and phosphorus management.

## Outcomes of Nutrient Management in Japan

In Japan, where various types of pollution intensified owing to rapid economic growth in the 1950s and 1960s, water pollution control earnestly began in the early 1970s. A system of areawide total pollutant load control was introduced in 1978 for eutrophication-prone enclosed seas, specifically Tokyo Bay, Ise Bay, Osaka Bay, and the Seto Inland Sea. The first phase in 1979 targeted chemical oxygen demand, and the fifth phase in 2001 added nitrogen and phosphorus. Organic pollutant and nutrient loads in these catchments peaked in the 1980s,^3^ and total nitrogen concentrations subsequently decreased (Figure 1). Nonetheless, red tides and hypoxia persisted, with Tokyo Bay recording 40–70 red tide events annually between 1980 and 2000 and approximately 30 in 2010 and the Seto Inland Sea having approximately 150 cases in the 1980s and approximately 100 in the 2000s.^3^ Simultaneously, fishery yields, particularly in the Seto Inland Sea, decreased from approximately 550 000 t annually in the 1980s to approximately 250 000 t in the 2000s. The production of farmed *nori* seaweed also substantially declined due to nitrogen deficiency, resulting in color fading, after 2000.^5^

**Figure 1:**
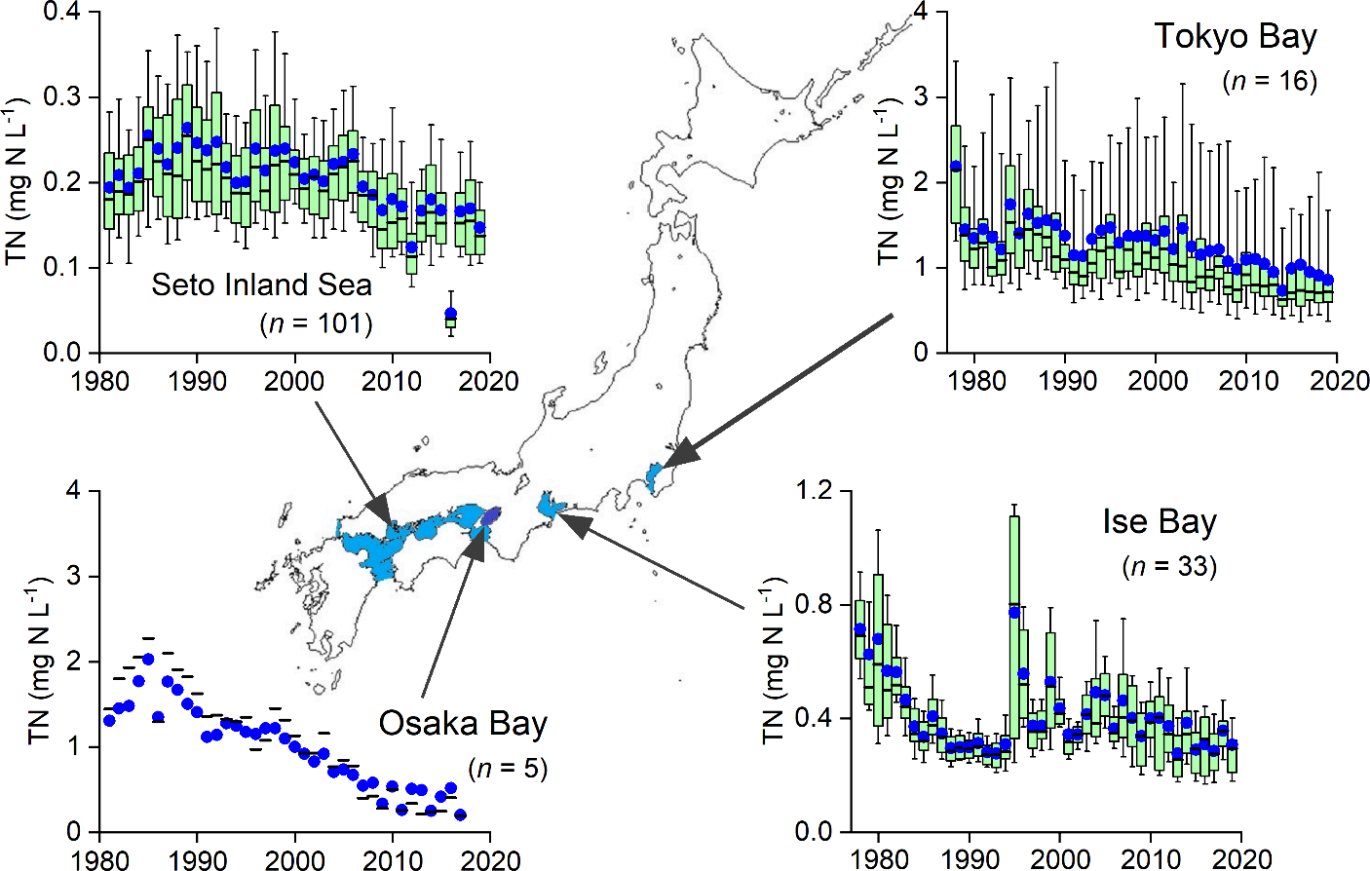
Surface layer total nitrogen (TN) concentrations in the four enclosed seas in Japan (data from ref 4). Blue circles denote the mean, boxes the 25th percentile, median, and 75th percentile, and whiskers the 10th and 90th percentiles. Note that only the mean and median are displayed for Osaka Bay.

## Challenge of Oligotrophication

Oligotrophication refers to a condition in which terrestrial nutrient inputs are reduced due to measures against human-induced eutrophication, while the nutrient demand of wild and farmed marine organisms exceeds the available supply, resulting in a nutrient deficit. Eutrophication and the resulting red tides cause hypoxia, which kills aerobic organisms. The frequent and widespread occurrence of these events causes mass mortality of marine organisms, and their decomposition further worsens hypoxia. Consequently, the original biodiversity and biomass decline. Human-induced loss of tidal flats and seaweed/seagrass beds, critical habitats for reproduction, impedes the recovery of biodiversity and biomass. Organic matter that accumulates in sediments during eutrophication can serve as a nutrient reservoir, potentially triggering red tides and hypoxia even after reductions in terrestrial nutrient inputs. The farming of lower-trophic-level organisms (e.g., *nori* and oysters) requires sufficiently high nutrient levels and may compete with other marine organisms for nutrients. These processes contribute to hysteresis, making it difficult to restore previous levels of biodiversity and biomass.^3^

Multiple factors influence the extent of oligotrophication caused by insufficient nutrient supply for primary producers such as phytoplankton, seaweed, and seagrass. These include not only nutrient concentrations but also oxygen levels, organic matter, turbidity, temperature, salinity, sediment conditions, food web dynamics, and ocean currents. Furthermore, human activities such as fishing, aquaculture, dredging, land reclamation, river improvement, and dam construction can cause nutrient imbalance and loss of tidal flats and seaweed/seagrass beds. Thus, the primary drivers and pressures of oligotrophication vary spatiotemporally, requiring localized assessments.

In Japan, oligotrophication requiring artificial nutrient inputs has been reported in Ise Bay, Mikawa Bay, the Seto Inland Sea, and the Ariake Sea.^6^ In Ise Bay and Mikawa Bay, at least 0.1 mg of dissolved inorganic nitrogen per liter is needed to support healthy *nori* growth.^7^

## Measures and Policy Innovations

The total pollutant loading control system in Japan includes setting total daily pollutant load targets, regulating industrial and domestic wastewater, upgrading sewage treatment plants, reducing excess fertilizer use, managing livestock waste, preserving and restoring tidal flats and seaweed/seagrass beds, monitoring water quality indicators, and reviewing the effectiveness of these measures. However, factors such as dam construction and water usage also influence nutrient delivery to coastal seas.^3^ To counter potential nutrient deficiencies, an active management strategy implemented in sewage treatment plants, particularly in the Seto Inland Sea watershed, is the relaxation of nitrogen and/or phosphorus removal during the winter months, when nutrient concentrations tend to be lower. In 2021, 47 treatment plants adopted this strategy.^6^ Evaluating the impact of such operations on nutrient budgets, water quality, fishery yields, and aquaculture remains essential.

## Outlook and Recommendations

Experience in Japan demonstrates that eutrophication prevention should be prioritized over reversal because ecosystems degraded by eutrophication require carefully balanced nutrient controls to avoid triggering oligotrophication. Consequently, Japan’s policies now reflect a shift toward flexible region-based nutrient management. One promising approach is Satoumi, an integrated coastal zone management concept aimed at restoring marine biodiversity and productivity through human interaction.^3,8^ Japan’s action plan highlights three key directions aligned with Satoumi to achieve clean and productive seas: reviewing the total pollutant loading control system and environmental standards, promoting adaptive wastewater treatment to supply nutrients when needed, and conserving/restoring tidal flats and seagrass beds.^2^

Future research should focus not only on preventing eutrophication but also on accelerating recovery from oligotrophication in collaboration with policy implementation. Advancing knowledge and technology to prevent mass mortality caused by hypoxia and to restore tidal flats and seaweed/seagrass beds is particularly important. The experience and knowledge gained, as well as technology developed, in Japan through the total nutrient loading control system can serve as a reference for other countries. We hope that through sustainable nutrient management, in collaboration with domestic and international efforts, clean and thriving seas will be ensured for future generations.
